# Weekend Cyclists vs. Regular Cyclists: Association of Physical Training Distribution on Performance, Cardiometabolic Parameters and Muscle Oxygen Saturation

**DOI:** 10.3390/sports14070281

**Published:** 2026-07-03

**Authors:** José González, Daniela Campos, Rafael Gutiérrez-Pino, Gerardo Weisstaub, Carlos Sepúlveda, Rodrigo Troncoso

**Affiliations:** 1 Nutrition and Physical Activity Research Laboratory (LABINAF), Institute of Nutrition and Food Technology, University of Chile, Santiago 7830490, Chile; jose.gonzalez@inta.uchile.cl (J.G.); ddcampos1@uc.cl (D.C.); 2 Innovation Center, Physiology Unit, MEDS Clinic, Santiago 7690000, Chile; rafael.gutierrez@meds.cl; 3 Nutritional Assessment and Body Composition Laboratory, Center for Research in Food Environments and Prevention of Chronic Diseases Associated with Nutrition (CIAPEC), Institute of Nutrition and Food Technology, University of Chile, Santiago 7830490, Chile; gweiss@inta.uchile.cl; 4 Institute of Health Sciences, University of O’Higgins, Rancagua 2841959, Chile

**Keywords:** weekend warrior, training frequency, cardiorespiratory fitness, muscular strength, body composition, NIRS, SmO_2_

## Abstract

Weekend cyclists are individuals who engage in vigorous physical activity only on weekends, as opposed to those who exercise regularly during the week. Research suggests that concentrating physical training on one or two days may benefit heart health and metabolism, similar to exercising regularly. However, it remains unclear whether weekend cyclists exhibit similar adaptations in metabolic, performance, and muscle oxygenation markers. The aim of this study is to compare cardiorespiratory fitness, body composition, cardiometabolic risk markers, muscle strength, and muscle oxygenation between cyclists who concentrated training on weekends and cyclists who distributed training across three or more days per week. In this study, we used an analytical, observational, non-experimental design that recruited 28 cyclists, divided into weekend cyclists (*n* = 14) and regular cyclists (*n* = 14). Body composition, blood tests, lower body strength, aerobic capacity, and muscle oxygen saturation were assessed. Results: Weekend cyclists exhibited lower VO_2_max (36.7 ± 3.9 vs. 48.9 ± 6.3 mL·kg^−1^·min^−1^), lower knee extension strength (3.16 ± 0.57 vs. 4.42 ± 0.83 Nm·kg^−1^), and reduced ΔSmO_2_ responses during exercise compared with regular cyclists (all *p* < 0.05). In addition, weekend cyclists presented higher body fat percentage (25.9 ± 3.8 vs. 17.2 ± 4.2%), greater waist circumference (90.5 ± 4.3 vs. 83.6 ± 5.1 cm), and lower HDL cholesterol levels (54.2 ± 8.4 vs. 64.1 ± 11.0 mg/dL). In conclusion, weekend cyclists have lower cardiorespiratory fitness, muscular strength, and reduced ΔSmO_2_ responses during incremental exercise, along with higher levels of visceral fat and triglycerides, compared to those who train three or more days a week. The distribution and frequency of training within their workout plans were associated with differences in cardiorespiratory fitness and cardiometabolic markers.

## 1. Introduction

Regular physical activity is part of a healthy lifestyle and has beneficial effects on people’s health. When practiced regularly, it contributes to a decrease in all-cause mortality, especially the risk of death from cardiovascular diseases [1,2] and cancer [2]. Furthermore, regular physical activity reduces the incidence of chronic non-communicable diseases such as type 2 diabetes, hypertension, and dyslipidemia [3]. Currently, the WHO recommends 150–300 min of moderate-intensity physical activity or 75–150 min of vigorous-intensity physical activity per week to benefit cardiovascular health, mental health, cognition, and sleep; improve body composition; and reduce all-cause mortality [4]. However, a deeper understanding of the dose–response relationship between exercise and health outcomes is essential to optimize the benefits of physical activity. The FITT principle (frequency, intensity, time, and type) provides a framework for exercise prescription, and variations in any of these components may lead to distinct physiological adaptations [5]. While total training volume is often emphasized, the distribution of that volume throughout the week may also influence training responses. Due to time constraints and lifestyle demands, some individuals meet current physical activity recommendations in only 1 or 2 days per week, a pattern commonly referred to as the “weekend warrior” [6,7,8]. In the context of cycling, individuals following this pattern will be referred to as “weekend cyclists” throughout this manuscript.

A study characterizing a group of British adults who cycled on weekends found that they had a slightly higher BMI than those who were regularly active and presented intermediate levels of risk factors [7]. Some surveys conducted in England and Scotland show that they had a 10–15% reduction in the risk of all-cause mortality compared to sedentary individuals [9]. Data from the US National Health Survey (1997–2013) showed that weekend cyclists have mortality rates similar to those of regularly active people, with a 10–30% reduction in the risk of all-cause, cardiovascular, and cancer mortality [10]. Furthermore, according to data from the US National Health and Nutrition Examination Survey (2007–2016), weekend cyclists reported the same benefits in reducing the visceral adiposity index as regularly active subjects [11]. However, a new study analyzing the same database revealed that weekend cyclists do not provide similar benefits in reducing cardiometabolic indices (waist circumference, height, triglycerides, and HDL-C) [12]. Recently, a study using accelerometer-derived physical activity data from the UK Biobank reported that individuals following a weekend warrior pattern exhibited cardiometabolic health characteristics similar to those of subjects who distributed their physical activity more evenly throughout the week [13]. Meyer et al. conducted a study assessing cardiorespiratory fitness in weekend cyclists, exposing 38 participants to different training distributions for 12 weeks. The results showed that both weekend cyclists and regular training improved cardiorespiratory fitness, as measured by maximum oxygen consumption (VO_2_max), compared with the control group, with no significant differences between the groups [14]. Furthermore, it has been reported that after 8 weeks of training, cyclists who trained twice a week were comparable to those who trained four times a week in terms of increases in VO_2_max, hemoglobin mass, and skeletal muscle oxidative capacity [15]. Evidence suggests that weekend cycling may provide cardiometabolic and cardiorespiratory benefits.

Cycling provides an interesting model for investigating this question because training load, intensity, and performance can be objectively quantified using power-based metrics and standardized physiological assessments. Furthermore, cycling performance depends strongly on cardiorespiratory fitness, muscular function, body composition, and metabolic efficiency, allowing a comprehensive evaluation of potential differences associated with training distribution. Despite the growing popularity of the weekend warrior pattern, little is known about how concentrating training into one or two days per week may be associated with these physiological characteristics in trained cyclists.

No studies have simultaneously evaluated cardiorespiratory fitness, body composition, muscle strength, cardiometabolic risk markers, and muscle oxygen saturation (SmO_2_) in weekend cyclists and regular cyclists. Muscle oxygen saturation (SmO_2_), assessed through near-infrared spectroscopy (NIRS), has emerged as a non-invasive tool to characterize local oxygen dynamics during exercise and may provide complementary information regarding physiological adaptations associated with different training patterns.

Based on the available evidence, we hypothesized that cyclists who distribute their training across three or more days per week would present more favorable cardiorespiratory fitness, body composition, muscle strength, cardiometabolic markers, and SmO_2_ responses than cyclists who concentrate most of their training volume on weekends. Therefore, this study aimed to compare cardiorespiratory fitness, body composition, cardiometabolic risk markers, muscle strength, and muscle oxygenation between cyclists who concentrated training on weekends and cyclists who distributed training across three or more days per week.

## 2. Materials and Methods

Study design. The study design consisted of a nonexperimental, observational, analytical model.

Sample size. The sample size calculation was based on VO_2_max. To calculate the sample size, an effect size of 1.1, a power of 0.80, and an α of 0.05 were used, requiring a minimum of 14 subjects per group (28 subjects) to detect significant effects. Stata version 17.0.0 was used for this calculation.

Participants. An open call was made through social networks to male athletes who practice cycling between 5–10 h per week and are aged between 30–40 years. Training records obtained from synchronized TrainingPeaks^®^ v.12 (TrainingPeaks LLC, Louisville, CO, USA and Strava^®^ (Strava Inc., San Francisco, CA, USA) accounts during the 8 weeks preceding enrollment were reviewed to verify training frequency, weekly cycling volume, and group allocation. Participants were classified as weekend cyclists when most of their weekly cycling volume was accumulated within one or two training days per week, whereas regular cyclists distributed a similar weekly volume across three or more days. Training Stress Score (TSS) and Intensity Factor (IF) were obtained from power-based training records according to the TrainingPeaks methodology. Because both platforms were synchronized using the same underlying training files, no relevant discrepancies in training frequency, weekly volume, or training load metrics were identified. The exclusion criteria included the use of illicit substances or substances prohibited by the World Anti-Doping Agency (WADA), active smoking, a history of alcohol abuse, and treatment for weight loss, hypertension, thyroid disorders, or other pharmacological conditions that could affect cardiometabolic outcomes. All participants signed an informed consent form in accordance with the Declaration of Helsinki, which was approved by the ethics committee of the Instituto de Nutrición y Tecnología de los Alimentos (INTA) under code N° 11/2023. Participants classified as weekend cyclists were assigned to the WW group (*n* = 14), whereas those classified as regular cyclists were assigned to the RT group (*n* = 14). (detailed in Table 1). All assessments were performed during a single laboratory visit in the morning. Participants were instructed to avoid vigorous exercise for at least 24 h, abstain from alcohol for 48 h and caffeine for 12 h, and arrive after an overnight fast.

Training Stress Score (TSS) and Intensity Index (IF). Both TSS and IF were obtained from power-based training records during the 8 weeks preceding enrollment. These metrics were calculated according to the TrainingPeaks methodology, in which TSS integrates exercise duration and intensity relative to functional threshold power (FTP), and IF represents the ratio of normalized power to FTP. All participants used power meters during their cycling training, and data were reviewed using the same calculation approach regardless of the training platform (TrainingPeaks). When data originated from Strava, TSS and IF values were verified using synchronized power files generated from the same cycling sessions.

Primary and Secondary Outcomes. The primary outcomes of this study comprised changes in cardiometabolic risk factors and performance-related variables. Secondary outcomes included anthropometric measurements and muscle oxygenation kinetics, both evaluated for their established influence on physical performance.

Anthropometric measurements, body composition, and blood sampling. Participants were instructed to attend the laboratory after an overnight fast of at least 10 h and to refrain from vigorous physical exercise for at least 24 h before testing. Body composition was assessed by dual-energy X-ray absorptiometry (DXA) using a Lunar IDXA (GE Medical Systems, Madison, WI, USA). Participants lie supine on the scanning table, with arms slightly abducted from the trunk. DXA provides quantitative measures of fat mass and fat-free mass (g) for the whole body and specific regions (arms, legs, and trunk) [16]. All scans were performed using the same DXA device and software version, General Electric software v.13.6, to ensure comparability, with minimal significant variability (MSV) at the 95% confidence level between two measurements of the same object or subject of 0.9% [17]. Blood samples were collected by venous puncture for fasting blood tests (glucose, cholesterol, HDL-C, VLDL-C, LDL-C, triglycerides, cortisol, and testosterone) performed at Vidaintegra laboratories.

Handgrip strength. Handgrip strength was measured via a Takei GRIP-D handgrip dynamometer (T.K.K.5401, Takei Scientific Instruments Co., Ltd., Tokyo, Japan). It was evaluated in the dominant extremity via the evaluation method recommended by the American Society of Hand Therapists, which consists of evaluation in a seated position with an elbow angle of 90°. Three runs of 5 s of isometric The average tension were performed [18]. of the three attempts was registered and is shown as the mean ± S.D. The familiarization for this test consisted of performing at least 3 submaximal attempts to get a feel for the isometric resistance of the grip. The coefficient of variation for handgrip was 5.4% based on a previous study [19].

Maximum voluntary isometric contraction (MVIC). To evaluate MVIC, participants performed a standardized cycling warm-up consisting of 10 min at 1 W·kg^−1^. This low-intensity workload was selected to increase muscle temperature and prepare the lower limbs without inducing fatigue. After this, single-knee extension was evaluated in a seated position at a 90-degree angle, with the horizontal axis comprising the femoral condyle used as a reference. For its execution, three 5 s maximum extensions were performed with 5 min rests between each repetition. This evaluation was performed for both the dominant and nondominant legs in a quadriceps table attached to a transducer [20]. The average of the three attempts was recorded and is presented as mean ± SD. Maximal isometric knee extension strength was normalized to body mass and expressed as Nm·kg^−1^. The familiarization for this test involved performing at least 5 submaximal attempts to better understand the isometric resistance. The coefficient of variation for maximal voluntary isometric contraction was 4.2% based on a previous study [21].

Cardiorespiratory Fitness. Cardiorespiratory fitness was assessed by measuring maximum oxygen uptake (VO_2_max) using the participant’s bicycle mount on a Tacx Neo Smart 2T Trainer (Model T2875; Garmin Ltd., Olathe, KS, USA). Gas exchange data were collected via an automated breath-by-breath system, PNOĒ^®^ ergospirometer (ENDO Medical, Palo Alto, CA, USA). The measuring instruments were calibrated before each test, and the necessary environmental adjustments were made. The incremental test consisted of a ramp test with steps of 1 min duration and increments of 20 watts. The test starts with 5 min in a resting state and then a warm-up of 5 min at an intensity of 80 watts [22]. Following the warm-up, the ramp test started at 100 W and increased by 20 W·min^−1^ until exhaustion. During the test, heart rate was monitored with a Garmin HRM-Dual™ Heart Rate Monitor (M/N A03711; Garmin Ltd., Olathe, KS, USA). The test ended when the participant could not sustain a cadence of 85–95 rpm and/or could not continue. Maximal effort was considered achieved when participants reached volitional exhaustion and met at least one of the following criteria: respiratory exchange ratio (RER) ≥1.10, heart rate within 10 beats·min^−1^ of the age-predicted maximum, or a plateau in oxygen uptake despite increasing workload. The first ventilatory threshold (VT1) was determined using the criteria of an increase in VE/VO2 with no increase in VE/VCO2 and the departure from linearity of VE. The second ventilatory threshold (VT2) was determined by using the criterion of an increase in both VE/VO2 and VE/VCO2. Two observers detected VT1 and VT2 according to the previously described criteria [23].

Muscle oxygenation. Two commercially available continuous-wave near-infrared spectroscopy devices Moxy Muscle Oxygen Monitor (Fortiori Design LLC, Hutchinson, MN, USA) were placed on the vastus lateralis of both the dominant and nondominant legs to measure muscle oxygen saturation (SmO_2_). To establish the baseline, the participants were in a seated position for 5 min before the ramp test [24]. The sensors were fixed with medical tape to avoid displacement and covered with compression clothing designed for cycling. SmO_2_ was assessed during the incremental test. Using the MOXY monitor platform, both device datasets were smoothed by calculating the 3 s moving average at 2 Hz.

Delta SmO_2_. It was calculated as the difference between resting SmO_2_ and the minimum SmO_2_ value reached during the incremental test. MOXY monitor placement followed manufacturer recommendations and was standardized across participants. Prior to placement, the skin was cleaned and shaved when necessary to optimize signal quality. Adipose tissue thickness over the vastus lateralis was measured using a skinfold caliper and evaluated according to the manufacturer’s guidelines for NIRS signal acquisition before sensor placement.

Statistical analysis. Data normality was assessed separately for each variable within each group using the Shapiro–Wilk test. Continuous variables are presented as mean ± standard deviation (SD). Between-group comparisons were performed using an independent Student’s t-test for normally distributed variables and the Mann–Whitney U test for non-normally distributed variables. Mean differences are reported together with their corresponding 95% confidence intervals (95% CI).

Effect sizes were calculated to complement *p*-value interpretation. Cohen’s d was used for parametric comparisons, whereas rank-biserial correlation (r) was calculated for non-parametric analyses. Correlation analyses were performed using Pearson’s correlation coefficient for normally distributed variables and Spearman’s rank correlation coefficient for non-normally distributed variables. Primary analyses were conducted using pooled data from both groups to explore associations across the entire study population. Additional within-group correlation analyses were performed separately in regular and weekend cyclists to evaluate the potential influence of group allocation on the observed associations.

Given the exploratory nature of the study, the relatively small sample size, and the risk of increasing type II error, no formal correction for multiple comparisons was applied. Therefore, the findings should be interpreted with appropriate caution, as the large number of comparisons may increase the risk of type I error. Statistical significance was set at *p* < 0.05. All analyses were performed using GraphPad Prism version 10.0 (GraphPad Software, Boston, MA, USA).

## 3. Results

Table 1 shows the baseline anthropometric, body composition, and training characteristics of the subjects according to their training distribution. Compared to regular cyclists, the weekend cyclist group included more overweight individuals (regular cyclists = 0/14, weekend cyclists = 10/14) with greater fat mass and waist circumference (*p* < 0.001). However, fat-free mass and appendicular skeletal muscle mass did not differ significantly between the two groups. Analysis of training characteristics revealed similar values for training stress score, intensity index, and hours of cycling per week in both groups. The regular cyclists accumulated more than twice the weekly elevation gain of weekend cyclists (*p* < 0.001) despite similar weekly training volume, training stress score, and intensity index.

When comparing cardiometabolic risk factors, weekend cyclists exhibited higher vLDL cholesterol concentrations, lower HDL cholesterol levels, and a higher visceral adiposity index than regular cyclists (*p* < 0.05). Triglyceride concentrations also tended to be higher in weekend cyclists, although this difference reached only borderline statistical significance (*p* = 0.050). No significant differences were observed in fasting glucose, blood pressure, cortisol, or testosterone concentrations (Table 2).

To compare muscle strength between weekend and regular cyclists, we assessed handgrip strength in the dominant hand and knee extension strength in both legs. The findings indicate that both groups have comparable handgrip strength. However, regular cyclists show greater knee extension strength in both legs than weekend cyclists (*p* < 0.001). Cardiorespiratory fitness results indicate that weekend cyclists have a lower VO_2_max and reach ventilatory threshold 1 (VT1) with lower VO_2_max and peak aerobic power (*p* < 0.001), with no difference in maximum heart rate compared to regular cyclists (Table 3).

**Figure 1:**
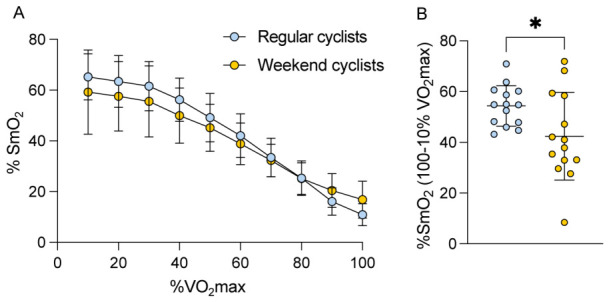
Muscle oxygen saturation (SmO_2_) responses during incremental exercise in regular and weekend cyclists. (**A**) SmO_2_ responses measured in the vastus lateralis during the incremental exercise test and expressed relative to the percentage of maximal oxygen uptake (%VO_2_max). Data are presented as mean ± SD. (**B**) ΔSmO_2_, calculated as the difference between resting SmO_2_ and the minimum SmO_2_ value reached during exercise. Individual values and group means ± SD are shown. * *p* < 0.05 versus regular cyclists (Student’s *t*-test).

Regular cyclists showed a greater ΔSmO_2_ response during the incremental exercise test than weekend cyclists (*p* < 0.05). Although SmO_2_ progressively declined with increasing exercise intensity in both groups, between-group differences appeared more evident at higher exercise intensities, with regular cyclists reaching lower minimum SmO_2_ values at peak exercise. These findings suggest distinct muscle oxygenation responses during incremental exercise according to training distribution (Figure 1).

**Figure 2:**
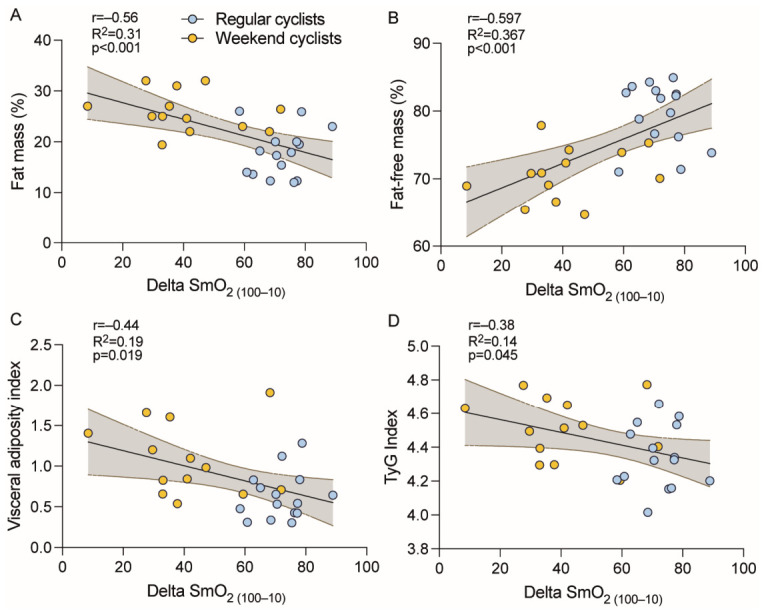
Pooled correlations between ΔSmO_2_ and body composition and metabolic indices. Correlations between ΔSmO_2_ and (**A**) fat mass, (**B**) fat-free mass, (**C**) visceral adiposity index, and (**D**) triglyceride-glucose index. Data from regular and weekend cyclists were pooled for the correlation analyses. Pearson’s correlation coefficients are shown as r, together with R^2^ and *p*-values. TyG: triglyceride-glucose index.

To determine whether body composition or metabolic indices are related to oxygen saturation, we examined correlations between muscle oxygen delivery (Delta SmO_2_) and fat mass, fat-free mass, the visceral adiposity index, and the TyG index. Fat mass was negatively correlated with ΔSmO_2_ (r = −0.56, R^2^ = 0.31, *p* < 0.01), whereas fat-free mass was positively correlated with ΔSmO_2_ (r = 0.60, R^2^ = 0.36, *p* < 0.001). In addition, visceral adiposity index (r = −0.44, R^2^ = 0.19, *p* = 0.019) and TyG index (r = −0.38, R^2^ = 0.14, *p* = 0.045) were negatively correlated with ΔSmO_2_ (Figure 2). These exploratory pooled correlations suggest associations between ΔSmO_2_ and body composition and metabolic characteristics. However, additional within-group analyses demonstrated that several of these associations were attenuated after stratification by training group (Supplementary Table S1), suggesting that some pooled correlations may partially reflect differences between groups.

**Figure 3:**
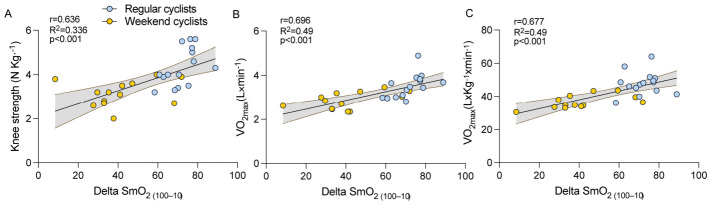
Pooled correlations between ΔSmO_2_, lower-limb strength, and cardiorespiratory fitness. Correlations between ΔSmO_2_ and (**A**) knee extension strength, (**B**) absolute VO_2_max, and (**C**) relative VO_2_max. Data from regular and weekend cyclists were pooled for the correlation analyses. Pearson’s correlation coefficients are shown as r, together with R^2^ and *p*-values.

To determine whether muscle strength and cardiorespiratory fitness were associated with muscle oxygen saturation responses, we examined pooled correlations between ΔSmO_2_, knee extension strength, absolute VO_2_max, and relative VO_2_max. ΔSmO_2_ was positively correlated with knee extension strength (r = 0.86, R^2^ = 0.73, *p* < 0.001), absolute VO_2_max (r = 0.70, R^2^ = 0.48, *p* < 0.001), and relative VO_2_max (r = 0.88, R^2^ = 0.77, *p* < 0.001) (Figure 3). Additional within-group analyses were performed to evaluate whether these associations were independent of training group allocation. Although several pooled associations were attenuated after stratification by group, the association between ΔSmO_2_ and knee extension strength remained significant among weekend cyclists (Supplementary Table S1). These findings suggest that some pooled correlations may partially reflect differences between groups, whereas the relationship between muscle oxygenation responses and lower-limb strength may persist at the individual level.

## 4. Discussion

This study investigated variations in cardiometabolic risk factors and performance-related variables as primary outcomes. Additionally, anthropometric measurements and muscle oxygenation were examined as secondary outcomes due to their influence on physical performance. The results revealed that weekend cyclists had higher BMI, fat mass, and waist circumference, but no significant differences in appendicular muscle mass. Regular cyclists exhibited greater lower limb strength, cardiorespiratory fitness, and greater ΔSmO_2_ responses than weekend cyclists. These results are consistent with those reported in database studies and cohort analyses comparing weekend cyclists, regularly trained athletes, and sedentary populations. The main study by O’Donovan and Dos Santos reported similar BMI for weekend cyclists and regularly active subjects [9,10]. The subjects assessed in these studies used self-reported physical activity data from large cohorts and were classified as weekend warriors (regardless of exercise type) if they met the WHO recommendation (physically active; ≥150 min/week of moderate activity or ≥75 min/week of vigorous activity) in 1 or 2 sessions per week or if they were regularly active in ≥3 sessions per week. Notably, our study focused on long-distance cyclists who cycled approximately 8 h/week, distributed across 1–2 sessions/week (weekend cyclists) or ≥3 sessions/week (regular cyclists).

Body composition analysis showed higher visceral fat in weekend cyclists than in regular cyclists, which differs from previous reports that found similar abdominal fat in weekend cyclists compared to regular cyclists [25]. Interestingly, no differences in lean mass were found between weekend and regular exercisers in our study. Furthermore, comparing visceral adiposity indices between the regular and occasional exercise groups revealed an increased VAI in weekend cyclists, contrasting with the findings of Wang et al., who reported no differences between the groups [11]. However, both groups were below the cutoff point [26]. Physical activity and adherence to recommendations act as protective factors, improving adipose tissue function at both the visceral and hepatic fat levels [27]. In this context, physical activity performed for one or two days could positively affect adipose tissue function. In relation to the above, studies evaluating the frequency of high-intensity interval training (2 and 3 times per week) demonstrated that participants experienced decreases in waist circumference and insulin concentrations and increases in VO_2_max. However, those training 3 times per week showed a greater reduction in body fat percentage, total cholesterol, and LDL cholesterol, as well as improvements in mental health components [28]. Similarly, another study evaluating the distribution of high-intensity interval training on 3 days (double intervals) and 6 days (single intervals) showed that both groups increased their VO_2_max, fat and carbohydrate oxidation, and endurance capacity [29]. Regarding other biochemical parameters, fasting glucose concentrations were similar between groups. In contrast, weekend cyclists presented a less favorable lipid profile, characterized by higher VLDL concentrations, lower HDL cholesterol levels, and a tendency toward higher triglyceride concentrations.

Despite these differences, mean lipid concentrations in both groups remained within clinical reference ranges: triglycerides were below the hypertriglyceridemia threshold (<150 mg/dL), and HDL cholesterol remained above cardiovascular risk values. Thus, weekend cyclists showed a less favorable cardiometabolic profile than those with overt dyslipidemia. Although the difference in triglyceride concentrations reached only borderline statistical significance (*p* = 0.050), the observed moderate-to-large estimated effect size should be interpreted cautiously because both the effect estimate and the *p*-value are based on the same relatively small sample. Consequently, these findings require confirmation in larger, adequately powered studies before definitive conclusions can be drawn.

An important finding of the present study was the substantial difference in weekly elevation gain between groups. Regular cyclists accumulated more than twice the weekly ascent of weekend cyclists despite similar weekly training volume, training stress score, and intensity index. Therefore, some of the observed differences in lower-limb strength, cardiorespiratory fitness, and cardiometabolic characteristics may reflect differences in climbing exposure rather than training frequency alone. Climbing requires sustained high-intensity efforts and greater lower-limb muscle recruitment, which may contribute to superior neuromuscular and aerobic adaptations [30,31]. Consequently, the greater weekly ascent accumulated by regular cyclists should be considered a potential contributor to the higher VO_2_max, ventilatory thresholds, and muscle strength observed in this group [32]. Because of the relatively small sample size and the substantial between-group difference in weekly elevation gain, we did not perform adjusted analyses for climbing exposure. Such models would likely have been statistically underpowered and may have introduced overadjustment, as weekly elevation gain represents an integral component of the habitual training pattern of regular cyclists rather than a clear independent exposure. Therefore, the present findings should be interpreted as reflecting the combined influence of training distribution and habitual climbing exposure. In addition to differences in triglyceride and cholesterol levels, both groups remained within the normal range. Taken together, these findings suggest that differences in training frequency, distribution, and climbing exposure were associated with differences in body composition and cardiometabolic characteristics.

The negative associations between ΔSmO_2_ and adiposity-related variables are physiologically plausible. Increased body fat and visceral adiposity have been linked to endothelial and microvascular dysfunction, reduced nitric oxide bioavailability, low-grade inflammation, impaired skeletal muscle oxidative capacity, mitochondrial dysfunction, and lower aerobic fitness, all of which may reduce muscle oxygenation during exercise [30,31,33]. These mechanisms may help explain the associations between ΔSmO_2_, body composition, and cardiorespiratory fitness. However, after stratification by training group, several pooled correlations were markedly attenuated or reversed in direction, suggesting that these associations were driven primarily by differences between regular and weekend cyclists rather than by consistent physiological relationships within each group. Therefore, these pooled correlations should be considered exploratory and interpreted with caution. Larger studies specifically designed to examine within-group associations are required to determine whether these relationships persist independently of training group allocation.

Handgrip strength did not differ between groups, which is expected given that cycling is primarily a lower-limb activity with limited upper-extremity strength demands. Handgrip mainly contributes to bicycle handling, steering, and postural stabilization, whereas propulsion depends largely on lower-limb power-generating muscles [32,34]. Thus, the absence of handgrip differences, together with the greater knee extension strength observed in regular cyclists, is consistent with the task-specific nature of cycling-related neuromuscular adaptations [34]. Greater maximum voluntary isometric knee extension strength in regular cyclists may also reflect differences in training characteristics or accumulated climbing exposure.

Furthermore, regular cyclists exhibited higher absolute and relative VO_2_max, peak aerobic power, and ventilatory threshold power outputs than weekend cyclists, which may be associated with differences in training distribution and weekly elevation gain. We also observed that regularly trained cyclists showed higher lower limb strength during the maximum voluntary isometric contraction test, a result that correlated positively with SmO_2_ and VO_2_max. Previous studies have also established a relationship between improved muscle strength and cardiorespiratory fitness and a lower risk of all-cause mortality [35].

Regular cyclists exhibited greater ΔSmO_2_ responses than weekend cyclists and demonstrated higher lower-limb strength and cardiorespiratory fitness. Because SmO_2_ reflects the balance between oxygen delivery and oxygen utilization within the exercising muscle, these findings should be interpreted as differences in muscle oxygenation responses rather than direct evidence of enhanced oxygen delivery, capillary density, mitochondrial content, or muscle oxidative capacity. Additional physiological measurements would be required to confirm the mechanisms underlying these observations [36,37,38,39]. An additional methodological consideration is that NIRS signal quality may be influenced by subcutaneous adipose tissue thickness, which can attenuate light penetration and affect the accuracy of muscle oxygenation measurements [40,41]. Although adipose tissue thickness at the measurement site was assessed using a skinfold caliper before sensor placement to ensure compliance with the manufacturer’s recommended limits, and all participants met these criteria, residual effects of adipose tissue on the NIRS signal cannot be completely excluded. Therefore, this factor should be considered when interpreting the observed between-group differences in ΔSmO_2_.

Additional within-group analyses demonstrated that several pooled correlations were attenuated after stratification by training group. Therefore, some of the observed associations between ΔSmO_2_, body composition, and cardiorespiratory fitness may reflect differences between regular and weekend cyclists rather than continuous physiological relationships. Nevertheless, the association between ΔSmO_2_ and knee extension strength remained significant among weekend cyclists, suggesting a potential link between local muscle oxygenation responses and neuromuscular performance.

From a practical perspective, distributing cycling training across three or more days per week may be associated with better cardiorespiratory fitness, lower-limb strength, and body composition than concentrating training into one or two sessions. However, weekend cyclists still accumulated substantial training volume and maintained generally healthy cardiometabolic profiles, suggesting that weekend exercise remains beneficial when regular training is not feasible. Future interventional studies should determine whether adding short weekday sessions, increasing climbing exposure, or including strength training provides additional benefits.

This study has several strengths; standardized laboratory techniques were used, and the cardiorespiratory, anthropometric, and strength profiles of both groups of cyclists were directly evaluated. Furthermore, SmO_2_ was incorporated into the analysis as a non-invasive marker of muscle oxygenation, providing complementary physiological information that may be useful when interpreting training responses. This is particularly relevant because SmO_2_ has received limited attention in studies examining training distribution patterns among cyclists. Data from a group of subjects who cycle with a high weekly volume (>8 h) but with a different training distribution are also included.

Several limitations should be acknowledged. First, the relatively small sample size may have limited statistical power for some outcomes, which does not allow for adjusting the analysis and makes this study exploratory. Second, the cross-sectional design precludes causal inference. Third, dietary intake, sleep habits, occupational activity, and other lifestyle factors were not controlled and may have influenced body composition and cardiometabolic variables. Fourth, training records were obtained from self-reported training platforms, which may introduce measurement variability. Fifth, self-selection bias cannot be ruled out, as participants were not randomly assigned to study groups. Sixth, the substantial difference in weekly elevation gain between groups may have acted as a confounding factor influencing physiological and cardiometabolic outcomes. Finally, in total, 51 statistical tests were performed across all evaluated outcomes, consisting of 44 unpaired Student’s t-tests for between-group comparisons and 7 correlation analyses. We acknowledge as a limitation that a high number of outcomes and correlations were evaluated relative to our sample size (n = 14 per group). Although between-group comparisons were strictly pairwise, testing multiple variables simultaneously increases the risk of Type I error inflation. Due to the exploratory nature of this study, no formal adjustments for multiple comparisons were applied to avoid an excessive inflation of Type II errors. Consequently, these findings should be interpreted with caution and considered as hypothesis-generating for future confirmatory research.

In this exploratory cross-sectional study, cyclists training three or more days per week showed greater cardiorespiratory fitness, lower-limb strength, cardiometabolic profiles, muscle oxygenation responses, and reduced fat mass and waist circumference than those concentrating training into one or two sessions. Regular cyclists had ~33% higher relative VO_2_max (48.9 vs. 36.7 mL·kg^−1^·min^−1^) and ~40% greater knee extension strength. However, causality cannot be inferred, and the higher weekly elevation gain in regular cyclists should be considered. Longitudinal studies are needed to clarify the independent effects of training distribution and climbing exposure.

## Figures and Tables

**Table 1:** Baseline characteristics of the participants.

Anthropometric Characteristics	Regular Cyclist (n = 14)	Weekend Cyclists (n = 14)	*p*-Value	[95% CI]	Cohen’s d
	Mean ± SD	Mean ± SD			
Age (years)	33.9 ± 3.3	35.4 ± 3.5	0.259	−1.17, 4.17	−0.44
Height (cm)	176.5 ± 6.3	174.1 ± 5.9	0.306	−7.13, 2.32	0.39
Weight (kg)	73.0 ± 7.4	78.0 ± 6.9	0.075	−10.60, 0.55	−0.70
BMI (kg·m^−2^)	23.4 ± 1.1	25.7 ± 1.9	0.004	−3.56, −1.17	−1.54
Fat mass (%)	17.2 ± 4.2	25.9 ± 3.8	<0.001	−11.70,−5.52	−2.15
Fat-free mass (%)	80.1 ± 4.2	70.8 ± 3.8	<0.001	6.24, 12.40	2.35
Waist circumference (cm)	83.6 ± 5.1	90.5 ± 4.3	<0.001	−10.60, −3.21	−1.46
Neck circumference (cm)	37.1 ± 1.5	37.96 ± 1.6	0.167	−2.03, 0.37	−0.54
ASMM (kg)	24.7 ± 2.5	23.6 ± 2.0	0.210	−2.88, 0.66	0.49
ASMI (kg/mt^2^)	7.90 ± 0.46	7.84 ± 0.70	0.811	−0.51, 0.41	0.09
Training characteristics					
Training stress score	650 ± 125	575 ± 94	0.081	−161, 9.95	0.69
Intensity index	0.76 ± 0.06	0.74 ± 0.07	0.263	−0.07, 0.02	0.43
Cycling per week (h)	8.5 ± 1.1	7.8 ± 0.9	0.090	−0.11, 1.44	0.66
Total ascent per week (m)	2350 ± 479	1137 ± 276	<0.001	903.02, 1522.28	3.04

Data are presented as mean ± SD. Mean differences, 95% confidence intervals (95% CI), and Cohen’s d were calculated as Regular cyclists minus Weekend cyclists. Positive values indicate higher values in regular cyclists, whereas negative values indicate higher values in weekend cyclists. A *p*-value < 0.05 was considered statistically significant. BMI: body mass index; ASMM: appendicular skeletal muscle mass; ASMI: appendicular skeletal muscle index.

**Table 2:** Comparison of cardiometabolic risk factors between regular cyclists and weekend cyclists.

Cardiometabolic Characteristics	Regular Cyclists (n = 14)	Weekend Cyclists (n = 14)	*p*-Value	[95% CI]	Cohen’s d
	Mean ± SD	Mean ± SD			
Fasting glucose (mg/dL)	91.1 ± 7.5	88.6 ± 4.0	0.282	−2.17, 7.17	0.42
Systolic blood pressure (mmHg)	127 ± 11.1	131 ± 10.0	0.335	−12.20, 4.30	−0.38
Diastolic blood pressure (mmHg)	77.3 ± 7.7	79.8 ± 8.1	0.408	−8.60, 3.60	−0.32
Cholesterol (mg/dL)	172 ± 34	180 ± 30	0.541	−32.70, 17.5	−0.25
HDL-C (mg/dL)	64.1 ± 11	54.2 ± 8.4	0.015	2.10, 17.69	1.01
LDL-C (mg/dL)	93.9 ± 31	106.6 ± 24	0.234	−34.10, 8.81	−0.46
vLDL-C (mg/dL)	14.2 ± 5.6	19.1 ± 6.9	0.049	−9.83, −0.01	−0.78
Triglycerides (mg/dL)	71.1 ± 28	95.6 ± 34	0.050	−48.99, −0.01	−0.79
Visceral adiposity index	0.64 ± 0.3	1.04 ± 0.5	0.011	−0.70, −0.10	−0.97
TyG index	8.01 ± 0.3	8.29 ± 0.4	0.071	−0.57, 0.03	−0.79
Cortisol (μg/dL)	12.6 ± 2.6	12.3 ± 2.8	0.768	−1.82, 2.43	0.11
Testosterone (ng/dL)	509 ± 118	631 ± 202	0.062	−250.00, 6.40	−0.74
Testosterone/cortisol ratio	0.043 ± 0.02	0.053 ± 0.02	0.197	−0.03, 0.005	−0.50

Data are presented as mean ± SD. Mean differences, 95% confidence intervals (95% CI), and Cohen’s d were calculated as Regular cyclists minus Weekend cyclists. Positive values indicate higher values in regular cyclists, whereas negative values indicate higher values in weekend cyclists. A *p*-value < 0.05 was considered statistically significant. HDL-C: high-density lipoprotein cholesterol; LDL-C: low-density lipoprotein cholesterol; vLDL-C: very-low-density lipoprotein cholesterol; TyG index: triglyceride-glucose index.

**Table 3:** Muscle strength and cardiorespiratory fitness.

Characteristics of Muscle Strength and Cardiorespiratory Fitness	Regular Cyclists (n = 14)	Weekend Cyclists (n = 14)	*p*-Value	[95% CI]	Cohen’s d
	Mean ± SD	Mean ± SD			
Handgrip strength (kg)	44.9 ± 10.6	45.6 ± 9.1	0.864	−8.31, 7.02	−0.07
Knee extension strength (Nm·kg^−1^)					
Dominant leg	4.42 ± 0.83	3.16 ± 0.57	<0.001	0.71, 1.81	1.79
Non-dominant leg	4.05 ± 0.64	3.00 ± 0.53	<0.001	0.59, 1.51	1.79
VO_2_max (L·min^−1^)	3.56 ± 0.53	2.86 ± 0.37	<0.001	0.34, 1.06	1.54
VO_2_max (mL·kg^−1^·min^−1^)	48.9 ± 6.30	36.7 ± 3.91	<0.001	8.10, 16.30	2.33
Peak power output (W)	381.8 ± 40	297.1 ± 31	<0.001	56.90, 112.00	2.37
Peak heart rate (beats·min^−1^)	188 ± 5.7	185 ± 12	0.375	−3.21, 9.21	0.33
Oxygen pulse (mL/beats)	18.92 ± 3.2	15.47 ± 2.1	0.002	1.37, 5.54	1.27
VT1 (L·min^−1^)	2.36 ± 0.22	2.00 ± 0.22	<0.001	0.19, 0.53	1.64
VT1 (mL·kg^−1^·min^−1^)	32.46 ± 3.5	25.72 ± 2.6	<0.001	4.44, 9.23	2.19
% VT1 at VO_2_max	70.3 ± 4.0	67.1 ± 6.7	0.140	−1.12, 7.50	0.58
VT1-power (W)	211 ± 28.3	167 ± 17.8	<0.001	25.50, 62.30	1.88
VT2 (L·min^−1^)	2.95 ± 0.30	2.44 ± 0.33	<0.001	0.27, 0.76	1.61
VT2 (mL·kg^−1^·min^−1^)	40.7 ± 4.8	31.2 ± 3.5	<0.001	6.22, 12.7	2.28
% VT2 at V̇O_2_max	83.7 ± 7.85	85.2 ± 4.30	0.535	−6.42, 3.41	−0.24
VT2-power (W)	303 ± 37	230 ± 30	<0.001	46.50, 99.20	2.19

Data are presented as mean ± SD. Mean differences, 95% confidence intervals (95% CI), and Cohen’s d were calculated as Regular cyclists minus Weekend cyclists. Positive values indicate higher values in regular cyclists, whereas negative values indicate higher values in weekend cyclists. A *p*-value < 0.05 was considered statistically significant. VT1: ventilatory threshold 1; VT2: ventilatory threshold 2.

## Data Availability

The data are available upon reasonable request to the corresponding authors. The data are not publicly available under Chilean data protection law (Law N° 21.719).

## Supplementary Materials

The following supporting information can be downloaded at: https://www.mdpi.com/article/10.3390/sports14070281/s1, Table S1: Within-group correlations between ΔSmO2 and physiological variables.
