# Supplementary material for: Weekend Cyclists vs. Regular Cyclists: Association of Physical Training Distribution on Performance, Cardiometabolic Parameters and Muscle Oxygen Saturation
Source: Sports (Basel). 2026 Jul 3;14(7):281. doi: 10.3390/sports14070281 (PMC13417159; doi:10.3390/sports14070281)
Supplement: Supplementary file 1 [file sports-14-00281-s001.zip › sports-4339390-supplementary.pdf]

Supplementary Table S1. Within-group correlations between ΔSmO_2 and physiological variables.

Variable	Regular cyclists (r)	p-value	Weekend cyclists (r)	p-value
Fat mass (%)	0.367	0.196	0.150	0.609
Fat-free mass (%)	-0.171	0.558	-0.220	0.451
Visceral adiposity index	0.457	0.100	0.169	0.563
TyG index	0.345	0.227	0.219	0.451
Knee extension strength	-0.045	0.879	0.656	0.011
VO_2max ($L \cdot min^{-1}$)	-0.234	0.420	0.062	0.834
VO_2max ($mL \cdot kg^{-1} \cdot min^{-1}$)	-0.074	0.802	-0.246	0.396

Supplementary Table S1. *Within-group Pearson correlation analyses between ΔSmO_2 and body composition, metabolic, strength, and cardiorespiratory fitness variables in regular and weekend cyclists.*
